# Venous Bicarbonate as a Prognostic Biomarker and Proposed Proxy for Vital Capacity to Be Used as an Eligibility Criterion in Amyotrophic Lateral Sclerosis Clinical Trials

**DOI:** 10.1002/brb3.70570

**Published:** 2025-05-18

**Authors:** Juliette Foucher, Therese Wellander, Anikó Lovik, Linn Öijerstedt, Alexander Juto, Fang Fang, Caroline Ingre

**Affiliations:** ^1^ Department of Clinical Neuroscience Karolinska Institutet Stockholm Sweden; ^2^ Department of Neurology, ME Neurology Karolinska University Hospital Stockholm Sweden; ^3^ Unit of Integrative Epidemiology, Institute of Environmental Medicine Karolinska Institutet Stockholm Sweden; ^4^ Methodology and Statistics Unit, Institute of Psychology Leiden University Leiden the Netherlands

**Keywords:** amyotrophic lateral sclerosis, biomarker, clinical trials, survival, venous bicarbonate

## Abstract

**Background:**

Clinical trials for people living with ALS (pALS) all list vital capacity (VC) as an important eligibility criterion. However, VC measures are challenging to perform among pALS, especially bulbar pALS. Additionally, since the disease rapidly changes, the VC can change in a short duration of time, making it unreliable.

**Objective:**

We aimed to investigate the association of venous bicarbonate with VC as an alternative criterion for trial eligibility. We also wanted to examine whether venous bicarbonate could be used as a prognostic biomarker for survival in ALS.

**Methods:**

We included pALS from the Swedish ALS/MND Registry between January 1, 2019 and July 31, 2022. pALS had to have serum bicarbonate values and ALSFRS‐R available close to diagnosis. Spearman correlations, Kaplan–Meier curves, and Cox proportional hazard regressions were used to assess the associations of venous bicarbonate with VC, other clinical characteristics, and survival.

**Results:**

Among the 117 pALS included in the study, we observed a negative correlation between venous bicarbonate and VC among spinal and bulbar pALS (*ρ* = −0.346, *p* = 0.002 and *ρ* = −0.367, *p* = 0.024, respectively). Venous bicarbonate negatively correlated with ALSFRS‐R (*ρ* = −0.377, *p* < 0.001), specifically among bulbar pALS (*ρ* = −0.595, *p* < 0.001), but positively correlated with KING's stage (*ρ* = 0.248, *p* = 0.007). High level of venous bicarbonate appeared to be associated with shorter survival.

**Conclusions:**

Venous bicarbonate appears to be a prognostic biomarker for survival among pALS. This cheap and easily accessible measure could potentially be an alternative for VC as an eligibility criterion in ALS trials.

## Graphical abstract

Graphical abstract, Venous bicarbonate as a prognostic biomarker and proposed proxy for vital capacity to be used as an eligibility criterion in amyotrophic lateral sclerosis clinical trials.

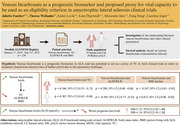

## 1 Introduction

Amyotrophic lateral sclerosis (ALS) is a fatal neurodegenerative disease, affecting both upper and lower motor neurons and resulting in progressive muscular paresis, causing death due to respiratory failure or cardiovascular events in most patients (Brown and Al‐Chalabi 2017, Larson et al. 2023). There is currently no disease‐modifying treatment and the people living with ALS (pALS) are confronted with a rapid progression of the disease without any effective therapy to reverse its fatal development (Feldman et al. 2022). The European incidence of ALS ranges from 2.1 to 3.8 per 100,000 person‐years (Longinetti and Fang 2019), but recent numbers in Sweden are higher and are estimated to around 4.0 per 100,000 person‐years (Longinetti et al. 2018, Imrell et al., 2024 May 22). In the Stockholm region, this translates to around 65 new pALS diagnosed each year (Svenska Neuroregister 2023). The large heterogeneity among pALS is seen both in the phenotypic expression and in progression rate (Al‐Chalabi et al. 2016). Most pALS are diagnosed with a spinal form of ALS, whereas one‐third of pALS first experience the loss of muscular function in the bulbar region, affecting the tongue, mouth, face, and pharynx (Feldman et al. 2022).

Respiratory failure, caused mainly by the weakness of the respiratory muscles, can present at any time during the disease, but typically occurs 3–5 years after diagnosis. Regardless of the time of onset of respiratory symptoms, ALS remains a fatal disease, pushed forward only by the use of ventilation support (Brown and Al‐Chalabi 2017). Many patients experience nocturnal hypoventilation symptoms first like orthopnea, leading to morning headaches or fatigue and sleepiness during daytime (Mirabile et al. 2024, Niedermeyer et al. 2019). Some pALS struggle to notice the progressive respiratory symptoms, since the symptoms are usually difficult to interpret. Thus, the assessment of respiratory function in ALS is fundamental to detect the symptoms at an early stage to be able to inform the patients and if needed initiate suitable aids, one being the initiation of non‐invasive ventilation (NIV) (Van Damme et al. 2024). The recent guidelines on clinical management of pALS by the European Academy of Neurology (EAN) now provide recommendations for respiratory insufficiency (Van Damme et al. 2024).

One of the most studied investigations of respiratory function is the vital capacity (VC), either measured through forced vital capacity (FVC) or slow vital capacity (SVC) (Niedermeyer et al. 2019, David and Sharma 2024). VC consists of the maximal volume of air that can be expired following maximum inspiration (Pereira 2020), FVC being measured after a pALS's exhale with maximum effort and speed, and SVC being measured after natural and unforced effort (Pereira 2020). It has been shown that FVC and SVC decrease with similar rate in all patients, both the pALS with bulbar and spinal onset, indicating a strong correlation and thus interchangeable clinically (Pinto and de Carvalho 2019). VC volume is measured in liters or expressed as a percentage of the predicted value for an individual, based on age, height, and weight, but does not consider site of onset (Goldman and Becklake 1959, van Damme et al. 2024). VC is the most important tool in the decision‐making process when deciding if a patient should be offered ventilation aid as NIV treatment (Wang et al. 2019).

In trials, the main variables considered for eligibility are El Escorial category, disease duration, and age (van Eijk et al. 2019 Jan 9). El Escorial criteria are mainly based on clinical findings although they appreciate results of electrophysiological studies, and can be subcategorized in definitive ALS, probable ALS, or probable and laboratory‐supported ALS (Verma 2021). Many ALS clinical trials have reported a threshold of respiratory function of VC ≥ 50% to ≥80% in order to qualify for the trial (Beaulieu et al. 2021) done to minimize the risk of death due to respiratory failure during the trial.

In 2024, 13 clinical trials were performed in Sweden, most of them randomized double‐blind placebo‐controlled trials evaluating disease progression and survival (National Library of Medicine 2023). Among the 11 trials run at the site in Stockholm (Alarcan et al. 2023), 8 trials requested a VC above a pre‐specified threshold in order for the patient to be eligible (National Library of Medicine 2023). Obtaining reliable VC values represents a challenge in ALS mainly for three reasons: First, bulbar weakness: spirometry is tiring and stressful, especially for bulbar pALS (Lechtzin et al. 2018), due to the more pronounced weakness in the facial muscles preventing them from performing the assessment in an effective manner due to air leaking when sealing around the mouthpiece of the spirometer. Second, diaphragm weakness: due to the weakness of the diaphragm muscles, the patients have a more superficial respiration with decreased capacity of expiration. Finally, spasticity: the spasticity of the respiratory muscles can impact the coordination of the upper respiratory muscles, resulting in lower spirometry measurements (Lechtzin et al. 2018, Sathyaprabha et al. 2010). These obstacles create unreliable measurements and often falsely low values (Lechtzin et al. 2018), and stress the need for alternative respiratory assessments (Murray et al. 2021), which would benefit both the bulbar pALS and the overall trial recruitment, resulting in faster recruitment and increased generalizability of trial findings (Lechtzin et al. 2018).

Blood gas measurements can be performed both from the artery (arterial blood gas, ABG) and from the vein (venous blood gas, VBG) and capture variations of the acid–base balance in the blood, providing key information for identifying underlying causes of the disrupted equilibrium (Hopkins et al. 2023). The acid–base homeostasis is composed of a respiratory and a metabolic component that compensate each other to maintain a pH between 7.35 and 7.45 (Hopkins et al. 2023). The pulmonary system affects the pH by modulating the partial pressure of arterial CO₂ (PaCO₂) through respiratory rate while the renal system, the primary modulator of the metabolic component, reabsorbs bicarbonate and excretes acids (Hopkins et al. 2023). Bicarbonate, a key buffer, plays a central role in maintaining pH and is influenced by the respiratory and renal function, as well as electrolytes imbalances and metabolic processes (Hopkins et al. 2023).

In ALS, loss of respiratory function over time causes hypoventilation and chronic respiratory acidosis due to the increase of PaCO₂ (Bartter 1963). This leads to a further raising of the plasma bicarbonate and a decrease in plasma chloride (Bartter 1963). Bicarbonate and chloride level variations have been explored in several studies and the advantages of the arterial blood gas over only VC measurements in pALS have been previously investigated (Manera et al. 2023; Hadjikoutis and Wiles 2001b; Alarcan et al. 2023).

A recent study showed an association between ABG parameters and FVC as well as between ABG and the ALS Functional Rating Scale‐Revised (ALSFRS‐R) in early progression of ALS disease (Alarcan et al. 2023). ALSFRS‐R is an instrument for evaluating the functional status of patients with Amyotrophic Lateral Sclerosis, it's revised version including respiratory function (Cedarbaum et al. 1999). This association was more clearly observed in pALS with spinal onset, among whom bicarbonate levels had a significant association with survival (Alarcan et al. 2023). The same study also demonstrated a correlation between ABG parameters and body mass index (BMI). Oxyhemoglobin, pO₂, and oxygen saturation was shown to correlate with BMI for the bulbar form whereas only pO₂ and hemoglobin for the spinal form (Alarcan et al. 2023). These results highlight the benefits of performing analysis through ABG instead of performing VC when the pALS are unable or it is difficult to perform a spirometry assessment. However, ABG measurement is an invasive intervention and must be performed by a doctor in some countries, while VBG can be drawn from an already existing venous blood line by a nurse or assistant nurse, thus less harmful for patients and less demanding for healthcare personnel.

The potential of VBG as a proxy of respiratory failure has been described in a few previous studies (Manera et al. 2023; Hadjikoutis and Wiles 2001b; Qureshi et al. 2008). A study of 23 patients with motor neuron disease (MND) showed that venous serum levels of bicarbonate were associated with respiratory muscle weakness, suggesting that bicarbonate levels could provide useful information on ALS prognosis (Hadjikoutis and Wiles 2001b). While many studies have explored alternatives to VC for trial inclusion, such as ABG, the potential of VBG and especially venous bicarbonate in clinical trial settings remains scarcely investigated.

The aim of this study was to investigate (1) the association of venous bicarbonate with VC in a Swedish cohort of pALS and (2) to determine whether venous bicarbonate alone could be used as a prognostic biomarker for survival in ALS.

## 2 Methods

### 2.1 Study Population

Our cohort included all pALS who received their diagnosis between January 1, 2019 and July 31, 2022, who had both VC assessment and VBG data available, spinal or bulbar onset and ALSFRS‐R measurements collected within 60 days from the first VC. Data were extracted from the Swedish MND Quality Registry (MNDreg) and medical records, which have previously been reported to include 99% of the MND patients in the Stockholm (Longinetti et al. 2018). The study was approved by the Swedish Ethical Review Authority (DNR 2017‐1895‐31/1), completed in accordance with the Helsinki Declaration and in respect of Good Clinical Practice, with all participants providing oral informed consent for the MNDreg.

### 2.2 Data Collection

MNDreg includes clinical data from pALS, at every clinical visit, from diagnosis to death. The following variables were retrieved from MNDreg: sex, date of symptom onset, place of onset, age at diagnosis, body mass index (BMI), total ALSFRS‐R score, date of NIV initiation, date of tracheostomy, and date of death. Missing data from MNDreg were complimented by reviewing medical records.

Each VC was matched with a venous bicarbonate value, an ALSFRS‐R, and a BMI measurement recorded within 60 days from the first VC value. The first set of matched parameters after diagnosis was defined as baseline values.

ALSFRS‐R slope (ΔALSFRS‐R) at the first clinical visit after diagnosis was calculated using the following formula: (48 minus ALSFRS‐R score at first visit after diagnosis)/(time from onset to diagnosis) (Kimura et al. 2006). KING's stage was determined using an algorithm including the ALSFRS‐R score (Balendra et al. 2014). KING's staging is a method of calculating clinical stages in ALS patients based on the number of CNS regions involved and requirement for gastrostomy or NIV (Balendra et al. 2019). Variables, including serum bicarbonate, sodium and VC, were collected from medical records, as was information on co‐morbidities and prescribed drugs to rule out conditions and interactions inducing metabolic alkalosis (corticosteroids and diuretics).

### 2.3 Statistical Analyses

Descriptive analysis including mean and standard deviation (SD) was used for normally distributed quantitative variables, whereas median and interquartile range (IQR) were calculated for non‐normally distributed quantitative variables such as time intervals.

All analyses were first performed for the entire study population and then separately for pALS with different sites of onset. Differences between spinal pALS and bulbar pALS were assessed using Student's *t* and Pearson's χ^2^ tests for quantitative and qualitative variables, respectively. Association between serum bicarbonate and clinical parameters was analyzed using Spearman's rank correlation coefficient (*ρ*).

For the pALS with multiple measures of venous bicarbonate and ALSFRS‐R within 60 days a repeated VC measure was performed, rate of decline was calculated by calculating the difference between the first and last measures, divided by the time interval between the two measures. It was done similarly with pALS having BMI values within 60 days of two different VC values. Associations between decline rates and bicarbonate were investigated using Spearman's rank correlation coefficient. This method allows us to assess for monotonous relationships between non‐normally distributed quantitative variables.

Overall survival of the pALS in relation to different levels of serum bicarbonate at baseline (in quartiles) was illustrated with Kaplan–Meier curves. Univariate Cox proportional hazard models were applied to assess the association of venous bicarbonate and clinical parameters with survival. They were presented as hazard ratio (HR) with 95% confidence interval (CI) of death. Independent variables included in the regression models were sex, age at diagnosis (above or below median value), site of onset, venous bicarbonate (divided into quartiles), VC, and ΔALSFRS‐R. Two multivariate analyses were conducted, called Multivariate Analysis A and Multivariate Analysis B, respectively. All factors with *p* < 0.2 in the univariate models were then included in the multivariable analyses. We conducted a Multivariate Analysis B representing a sensitivity analysis to Multivariate Analysis A, removing VC from the analysis, due to the correlation of VC and venous bicarbonate as well as VC and site of onset, shown in Section 3. Cox regression analysis was done in the same manner to predict NIV start.

All data were analyzed using IBM SPSS Statistics version 29.0.1.0–171. Bonferroni correction for multiple testing was applied with 29 tests considered. *p* values < 0.001 were considered as statistically significant.

## 3 Results

### 3.1 Participant Characteristics

**FIGURE 1:**
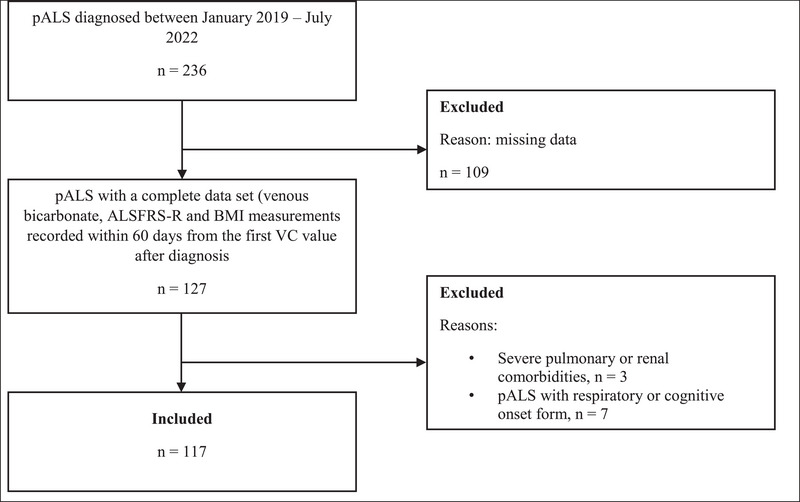
Flow chart of participant inclusion. pALS: people living with ALS; ALSFRS‐R: amyotrophic lateral sclerosis functional rating scale revised; BMI: body mass index; VC: vital capacity.

Between January 1, 2019 and July 31, 2022, 236 patients were diagnosed with ALS, adhering to the revised El Escorial criteria for clinically definite, probable, or possible ALS. A total of 117 pALS were included in this study according to the exclusion and inclusion criteria described in Section 2.1 (Figure 1).

The characteristics of the study participants are summarized in Table 1 and proportions of males and females across subgroups were nearly equal (Table 1). A total of 79 pALS had a spinal onset and 38 a bulbar onset. Among these patients, 16 pALS started NIV treatment and no pALS had a tracheostomy at baseline or anytime during follow‐up.

**TABLE 1:** Characteristics of the study participants.

Characteristics	All pALS (*n* = 117)	Spinal pALS (*n* = 79)	Bulbar pALS (*n* = 38)
Sex, *number (%)* Female Male	63 (53.8) 54 (46.2)	40 (50.6) 39 (49.4)	23 (60.5) 15 (39.5)
Age at diagnosis, *mean (SD)*	65.2 (10.8)	64.0 (11.0)	67.9 (10.1)
Diagnostic delay, *median months (IQR)*	12 (7–17)	12 (7–19)	12 (7–16)
KING's stage, *number (%)* 1 2 3 4A 4B	24 (20.5) 35 (29.9) 50 (42.7) 3 (2.6) 5 (4.3)	11 (28.9) 10 (26.3) 13 (34.2) 1 (2.6) 3 (7.9)	13 (16.5) 25 (31.6) 37 (46.8) 2 (2.5) 2 (2.5)
VC value at baseline in %, *mean (SD)*	75.4 (22.5)	79.7 (21.5)	66.4 (22.3)
Days between diagnosis and VC measure, *median days (IQR)*	65 (25–165)	69 (21–161)	57 (28–189)
Disease duration at VC measure, *median months (IQR)*	(15 (11–24)	15 (11–25)	16.5 (10–21)
Venous bicarbonate at baseline in *mmol/L*, *mean (SD)*	24.8 (2.3)	24.5 (1.6)	25.5 (3.3)
ALSFRS‐R score at baseline, *mean (SD)*	33.9 (8.4)	33.3 (8.8)	35.2 (7.5)
ΔALSFRS‐R at baseline, *mean (SD)*	1.05 (0.97)	1.01 (0.81)	1.14 (1.25)

*Note*: Numerical variables are presented as mean (standard deviation), categorical variables are presented as *n* (%), and time intervals are presented as median (interquartile range). VC, venous bicarbonate, ALSFRS‐R, and ΔALSFRS‐R values are the first measures recorded after diagnosis.

Abbreviation: ALSFRS‐R, Amyotrophic Lateral Sclerosis Functional Rating Scale‐Revised; BMI, body mass index; NIV, non‐invasive ventilation; pALS, people with ALS; VC, vital capacity.

### 3.2 Blood Values and Clinical Parameters

At baseline, venous bicarbonate levels were higher among pALS with bulbar onset compared to those with spinal onset (mean 25.53 mmol/L for bulbar, 24.51 mmol/L for spinal, *p* = 0.026) (Table 1), and VC measures were lower (mean 66.4% for bulbar, 79.7% for spinal, *p* = 0.03) (Table 1). However, the time between diagnosis and first VC measure did not differ between the two groups.

The mean serum bicarbonate was considered normal (22–27 mmol/L) and not statistically different between pALS with use of prescribed drugs (*n* = 44), such as corticosteroids or diuretics, and pALS without (mean = 24.7 mmol/L; SD = 2.0 and mean = 25.0 mmol/L; SD = 2.5; respectively).

The use of NIV was higher among the bulbar patients than spinal (24% vs. 9%), and they also had a shorter overall survival (mean of 27 months vs. mean of 35 months) (Table S1). No statistically significant differences were found between male and female pALS regarding VC, venous bicarbonate, nor ALSFRS‐R score (Table S2).

### 3.3 Correlation Between Venous Bicarbonate and FVC at Baseline

A moderate negative correlation (*ρ* = −0.345, 95% CI [−0.50 to −0.17]) was observed between venous bicarbonate and VC (Table 2). This was similar between spinal and bulbar pALS (*ρ* = −0.346, 95% CI [−0.53 to −0.13] and *ρ* = −0.367, 95% CI [−0.62 to −0.04], respectively).

**TABLE 2:** Spearman's correlation coefficients between venous bicarbonate and clinical parameters.

	All pALS (*n* = 117)	Spinal pALS (*n* = 79)	Bulbar pALS (*n* = 38)
VC	−0.345 [−0.50 to −0.17]	−0.346 [−0.53 to −0.13]	−0.367 [−0.62 to −0.04]
ALSFRS‐R	−0.377 [−0.53 to −0.21]	−0.288 [−0.48 to −0.06]	−0.595 [−0.77 to −0.33]
ΔALSFRS‐R	0.054 [−0.13 to 0.24]	−0.20 [−0.25 to 0.21]	0.221 [−0.11 to 0.51]
BMI decline	−0.009 [−0.41 to 0.40]	−0.095 [−0.54 to 0.39]	0.414 [−0.62 to 0.92]
KING's stage	0.248 [0.06 to 0.42]	0.216 [−0.01 to 0.42]	0.317 [−0.01 to 0.58]
Age at diagnosis	0.220 [0.03 to 0.39]	0.217 [−0.01 to 0.42]	0.176 [−0.16 to 0.47]
Disease duration	−0.059 [−0.24 to 0.13]	0.017 [−0.21 to 0.24]	−0.191 [−0.48 to 0.15]
Survival time	−0.257 [−0.45 to −0.03]	−0.257 [−0.50 to 0.03]	−0.209 [−0.54 to 0.18]
Time from the moment venous bicarbonate was collected to time of death	−0.356 [−0.54 to −0.14]	−0.240 [−0.49 to 0.04]	−0.584 [−0.79 to −0.26]
	**NIV pALS** (*n* = 16)	**NIV pALS** (*n* = 7)	**NIV pALS** (*n* = 9)
Time from the moment venous bicarbonate was collected to time NIV was initiated	−0.06 [−0.55 to 0.46]	0.356 [−0.56 to 0.88]	−0.190 [−0.77 to 0.56]

*Note*: Correlation coefficients are presented using Spearman's correlation coefficients (*ρ*, [95% CI]) and are compared for the full study population as well as for the spinal and the bulbar onset form.

Abbreviation: ΔALSFRS‐R, rate of functional decline over time; BMI, body mass index; NIV, non‐invasive ventilation; pALS, people living with ALS; VC, vital capacity.

### 3.4 Correlation Between Venous Bicarbonate and Clinical Parameters at Baseline

Venous bicarbonate was moderately negatively correlated with ALSFRS‐R (*ρ* = −0.377, 95% CI [−0.53 to −0.21]) and weakly positively with the age at diagnosis (*ρ* = 0.220, 95% CI [0.03–0.39]) (Table 2). The negative correlation with ALSFRS‐R was much stronger among bulbar pALS, compared to spinal pALS (*ρ* = −0.595, 95% CI [−0.77 to −0.33] and *ρ* = −0.288, 95% CI [−0.48 to −0.06], respectively). Venous bicarbonate also had a weak positive association with KING's stage (*ρ* = 0.248, 95% CI [0.06‐0.42]) at baseline.

### 3.3 Correlation Between Venous Bicarbonate and BMI

Venous bicarbonate increase rate moderately strongly correlated with BMI decline among pALS (*ρ* = −0.610, 95% CI [−0.82 to −0.24]). When comparing the onset groups, bulbar pALS had a much stronger negative correlation compared to spinal pALS (*ρ* = −0.812, 95% CI [−0.98 to −0.03] for bulbar, *ρ* = −0.547, 95% CI [−0.83 to −0.05] for spinal). That correlation, however, was not seen between baseline bicarbonate and BMI decline.

### 3.4 Risk of Death

**FIGURE 2:**
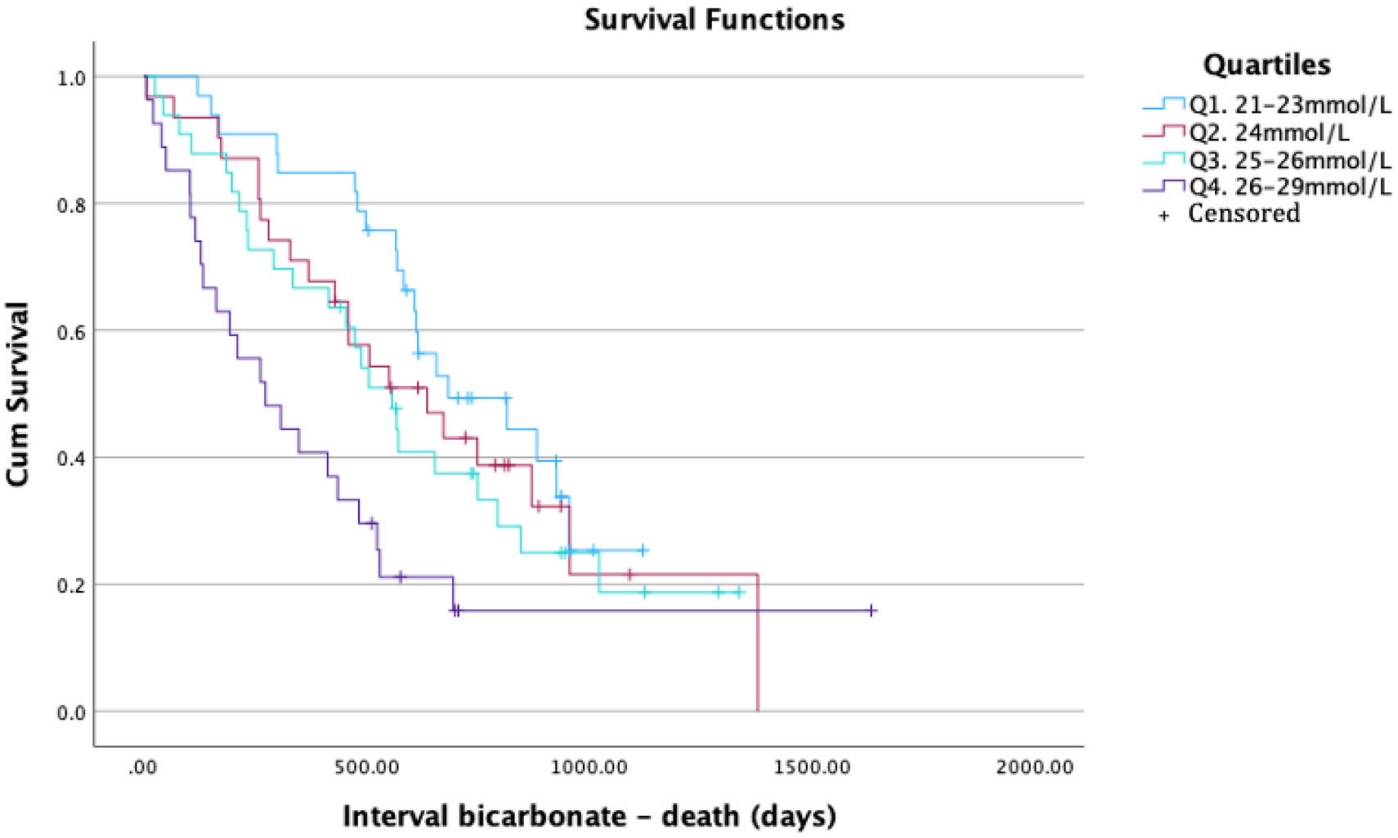
Kaplan–Meier analysis on overall survival, with pALS stratified by venous bicarbonate quartiles. Follow up: diagnosis to death or October 2023. Pairwise comparisons: Q1 versus Q2 (*p* = 0.370); Q1 versus Q3 (*p* = 0.108); Q1 versus Q4 (*p* < 0.001); Q2 versus Q3 (*p* = 0.520); Q2 versus Q4 (*p* = 0.038); Q3 versus Q4 (*p* = 0.073).

The Kaplan–Meier curve (Figure 2) suggested worse survival in relation to increasing levels of venous bicarbonate (*p* = 0.009). The fastest decline of survival was noted for pALS with the highest quartile of serum bicarbonate (≥26 mmol/L) (Figure 2).

In the univariable survival analysis (Table 3), four variables showed an association with survival at a cut‐off level of *p* < 0.2 as described in Methods. Higher level of serum bicarbonate, higher ΔALSFRS‐R, bulbar onset, and lower level of FVC were associated with higher risk of death (*p* = 0.002, *p* = 0.034, *p* = 0.139, and *p* < 0.001, respectively). These associations were also observed in the multivariable analysis (Multivariable Analysis A, Table 3), except for the site of onset where we observed an HR of 0.91 (0.54–1.55) which was not statistically significant (*p* = 0.736) and for venous bicarbonate with a HR of 1.07 (0.99–1.16) (*p* = 0.102) (Table 3). As we hypothesize that venous bicarbonate may act as a proxy for VC in the context of survival prediction, we also ran a multivariable analysis without VC (Multivariable Analysis B, Table 3): interestingly, we observed an HR of 1.12 [1.04–1.20] (*p* = 0.004) for venous bicarbonate, an HR of 1.25 [1.02–1.55] (*p* = 0.035) for ΔALSFRS‐R, and an HR of 1.36 [0.84–2.22] (*p* = 0.208) for site of onset. Additionally, we observed that including both VC and site of onset in the model caused multicollinearity, resulting in the drastic shift in HR between univariate model, model B versus model A as shown in Table 3.

**TABLE 3:** Factors influencing survival following an ALS diagnosis.

	Univariable analysis		Multivariable analysis A		Multivariable analysis B	
	HR [95% CI]	*p* value	HR [95% CI]	*p* value	HR [95% CI]	*p* value
Venous bicarbonate	1.13 [1.05–1.22]	0.002	1.07 [0.99–1.16]	0.102	1.12 [1.04–1.20]	0.004
VC	0.97 [0.96–0.98]	< 0.001	0.97 [0.96–0.99]	< 0.001		
ΔALSFRS‐R	1.25 [1.02–1.55]	0.034	1.31 [1.06–1.62]	0.014	1.25 [1.02–1.55]	0.035
Onset form	1.43 [0.75–1.42]	0.139	0.91 [0.54–1.55]	0.736	1.36 [0.84–2.22]	0.208
Sex	0.90 [0.58–1.40]	0.647				
Age at diagnosis	1.31 [0.84–2.04]	0.231				

*Note*: Uni‐ and multivariable Cox proportional hazards model for parameters measured at baseline. Results are presented as hazard ratios (95% confidence interval) and *p* value. Statistical significance was set at 0.2 for univariable analysis.

Abbreviation: ALSFRS‐R, Amyotrophic Lateral Sclerosis (ALS) Functional Rating Scale‐Revised; ΔALSFRS‐R, rate of functional decline over time; HR, hazard ratios; VC, vital capacity.

While venous bicarbonate was associated with a higher risk of death among both spinal and bulbar pALS (HR = 1.21, 95%CI [1.02–1.40], *p* = 0.025 for spinal pALS and HR = 1.14, 95%CI [1.03–1.26], *p* = 0.008 for bulbar pALS), the difference in HRs was not statistically significant.

### 3.5 Risk of NIV

The results of the univariable analysis showed that venous bicarbonate, FVC, and sex had a statistically significant association with the risk of NIV use (HR = 1.19, 95%CI [1.00–1.41] and *p* = 0.047, HR = 0.97, 95%CI [0.94–0.99] and *p* = 0.042, HR = 0.46, 95%CI [0.16–1.33], *p* = 0.15 respectively) (Table S3). We observed similar, although not statistically significant, associations in the multivariable analysis considering venous bicarbonate, VC and sex (HR = 1.14, 95%CI [0.96–1.35] and *p* = 0.148, HR = 0.97; 95% CI[0.94–1.00] and *p* = 0.086, and HR = 0.42, 95%CI [0.14–1.30] and *p* = 0.132 respectively) (Table S3). The Kaplan–Meier curve supports the relation of higher venous bicarbonate levels and sooner NIV initiation (Figure S1).

## 4 Discussion

In this study, we investigated the association between venous bicarbonate and VC, and whether venous bicarbonate could represent an accessible and less discriminant alternative to VC for trial eligibility, as well as the robustness as a prognostic marker. We observed that venous bicarbonate was indeed correlated to VC and that elevated bicarbonate measured in blood at the time around diagnosis was associated with shorter survival.

At baseline, mean venous bicarbonate levels were significantly higher among the bulbar pALS, confirming previously reported findings for ABG (Michels et al. 2022). To our knowledge, this is the first time a difference between bulbar and spinal pALS is described for VBG, a significantly more accessible method. Michels et al. found that bulbar onset pALS have elevated arterial bicarbonate (mean 27.6 mmol/L for bulbar and 25.4 mmol/L for spinal, *p* = 0.048) and PaCO_2_ (mean 42 mmHg for bulbar and 37 mmHg for spinal *p* = 0.028) compared to spinal onset pALS, which is in accordance with our observations (Michels et al. 2022). These results highlight a common acidosis phenomenon that seems to be more accentuated among bulbar pALS. A potential cause could be upper airway obstruction, which occurs in greater scale among bulbar pALS and could be due to muscular weakness in the pharyngeal and the laryngeal muscle, or to excessive mucal formation, leading to a more superficial respiration and a respiratory acidosis with increased bicarbonate levels (Hadjikoutis and Wiles 2001a). Blasco et al. did not note any difference in the levels of arterial bicarbonate between their onset groups, however their diagnostic delay was overall shorter, indicating perhaps a less significant muscle dysfunction and, therefore, a lower degree of acidosis (Alarcan et al. 2023)

Additionally, pALS with bulbar onset had significantly lower VC values compared to the spinal group, an observation supported by the literature. In previous studies, Michels et al. and Blasco et al. reported a similar pattern with FVC, observed in 55% to 90.2% of bulbar pALS and 71% to 93.9% of spinal pALS (*p* < 0.001 and *p* = 0.11, respectively) (Alarcan et al. 2023, Michels et al. 2022). This may reflect a real difference in respiratory function between the two groups as the bulbar phenotype has been characterized as more aggressive by numerous studies with a median survival of 2.0 years post‐symptom‐onset, and consequently an earlier decline of respiratory function (Czaplinski et al. 2006; Chiò et al. 2011). However, it might as well be an effect of involuntary glottic closure during forced expiration caused by facial dysfunction and difficulties forming a tight lip‐seal around the tube (Michels et al. 2022). Therefore, interpreting the results of the bulbar group presents a greater challenge, as the FVC values provided might be falsely low.

Higher levels of baseline venous bicarbonate were associated with lower VC, which is in accordance with the results by Qureshi et al. (2008). In their study, the correlation was, however, not done punctually for baseline measurements, but longitudinally over a period of 4 years. For each unit increase in serum bicarbonate, the slope of %VC decreased by 0.34 ± SE0.04 (*p* = 0.002). Among the studies on ABG, similar correlations between arterial bicarbonate at diagnosis and FVC was found by Manera et al. (*r* = −0.345 *p* < 0.001) as well as Blasco et al. (Alarcan et al. 2023). However, in the study by Blasco et al., when comparing the differences of place of region, the spinal pALS had the highest correlation coefficient (*r* = −0.467, *p* < 0.001 for spinal; *r* = −0.263, *p* = 0.013 for bulbar), a difference we did not find in our cohort. This discrepancy could be explained by the difference in baseline bicarbonate levels between our groups, potentially influencing the observed association.

Increasing venous bicarbonate was also found to correlate with decreasing ALSFRS‐R. Manera et al. observed the same correlation for arterial bicarbonate in their cohort of 488 pALS but only looking at ALSFRS‐R respiratory subscore (*ρ* = −0.290, *p* <0.001), evaluated by three out of the twelve ALSFRS‐R questions (Cedarbaum et al. 1999). Blasco et al. found a similar relationship in their cohort of 302 pALS but only in the spinal onset group (*ρ* = −0.34, *p* = 4.9 × 10^−6^) (Alarcan et al. 2023). This contrasts once again to our findings for the bulbar group, which exhibited a stronger correlation rate for ALSFRS‐R score. Moreover, a higher bicarbonate increase rate was associated with a greater BMI decline rate, a correlation that to our knowledge never has been reported before. In a study from 2023, Manera et al. investigated serum chloride, a proven indicator of chronic respiratory acidosis, (Manera et al. 2023), and found that lower serum chloride was correlated to greater weight loss prior to the time of diagnosis, which is consistent with our results. Although also considered as prognostic markers, BMI and ALSFRS‐R capture other aspects of disease progression compared to VC, but the correlation with bicarbonate reinforces its association with survival.

KING's stage was found to correlate with bicarbonate levels (*ρ* = 0.248, 95% CI [0.06‐0.42]), which to our knowledge has not been described before. KING's staging gives an indication of the anatomical spread of the disease as well as respiratory involvement and is thus, similarly to the other prognostic markers, a marker of bicarbonate's association with ALS progression (Fang et al. 2017).

Through both uni‐ and multivariable Cox regression models, we observed an association between higher baseline venous bicarbonate and higher risk of death. This is consistent with the results of Qureshi et al., who in their prospective study found an even higher association (HR = 1.47 *p* = 0.006), a difference potentially explained by their bigger sample size (*n* = 501) (Qureshi et al. 2008) This supports the physiological phenomenon when examining pALS, the progressive respiratory decompensation causes chronic respiratory acidosis resulting in an increase of serum bicarbonate levels. Overtime, the decompensation process reaches a critical point resulting in a continuous elevation of the acidosis, the most common cause of death among pALS. As our survival analysis suggests, pALS with baseline bicarbonate above 26 mmol/L exhibited poorer survival rates. Interestingly, this relates also to the cut‐off values of bicarbonate investigated by Manera et al., predicting NIV initiation and survival. A significant decline in FVC was recorded when arterial bicarbonate exceeded 26 mmol/ L (Manera et al. 2020). FVC and survival are tightly associated which could explain the concordance between both observations (Pirola et al. 2019)

The bulbar and spinal groups did not display any significant difference for risk of death with increasing bicarbonate. Blasco et al. showed that arterial bicarbonate was only significantly associated with survival in the spinal group, not the bulbar (Alarcan et al. 2023), a discrepancy that might be explained by their bigger cohort. We observed both in the univariable and multivariable Cox hazard model statistically significant relationships between venous bicarbonate and the risk of NIV use (Table S3). In the study conducted by Manera et al. including 760 pALS, serum chloride was observed to be highly predictive for time to NIV (HR = 0.67, IQR 0.46–0.97, *p* = 0.033) in the multivariable Cox regression analysis (Manera et al. 2023) This suggests that ion levels could be part of the decision making for NIV initiation, but further evaluation is needed.

The sensitivity analysis displayed in Table 3 highlights the issue of using VC and site of onset in the same survival model. As bulbar onset is associated with poor VC, it is not only discriminative for bulbar onset pALS as a trial eligibility criterion but also affects the validity of any statistical model that uses both VC and site of onset as predictors.

Our study is one of the first attempts to describe venous bicarbonate's correlation with VC and survival and should be regarded as a step toward more inclusive considerations for clinical trial eligibility (Hadjikoutis and Wiles 2001b, Qureshi et al. 2008) Distinctive analysis for bulbar and spinal pALS were notably conducted, making our study the first, to our knowledge, to examine the differences between onset forms for this specific blood parameter. Additionally, our study was not performed in the setting of a clinical trial, as previously done, and sensitivity analysis was done to detect the eventual effect of medication such as corticosteroids or diuretics (Qureshi et al. 2008). These two elements allowed us to avoid excluding pALS based on clinical trial exclusion criteria or medication use, making our cohort even more representative of the general pALS population. Furthermore, the prognostic feature of venous bicarbonate was assessed using several progression markers, strengthening the results observed.

However, we acknowledge several limitations. First, the ratio of affected males to affected females approaches 2:1 in the general ALS population whereas it was closer to 1:1 in our cohort (53.8% women and 46.2% men) (Brown and Al‐Chalabi 2017) Given that the impact of sex on VC measurements and bicarbonate levels was considered as non‐significant (mean VC 74.1% for male 76.5 for female *p* = 0.58 and mean venous bicarbonate 24.7 mmol/L for male and 25.0 mmol/L for female *p* = 0.47), our result could still be applicable in the general ALS population. Second, the retrospective nature of our study also had some limitations. Venous bicarbonate was not recorded at the same time as VC (mean 16 days, SD = 17 days). Both parameters can be sensitive to fluctuations due to various factors, and even the smallest changes, can have an impact. One way to mitigate the variability of venous bicarbonate in the future and in a potential clinical trial setting would be to perform three venous bicarbonate measurements during the trial screening visit (at arrival, after 1 h, and after 2 h) and calculate a median value. The lack of data also impacted the sample size and the significance, particularly for the subgroup of pALS with respiratory form or ALS‐FTD. Given the relatively small size of our cohort, the statistical power can be considered limited. Moreover, multiple drugs and assisting tools can potentially influence acid–base homeostasis, steroids and diuretics represent just a minor selection. Sedatives, anti‐anxiety medication, or opioids are other examples of drugs potentially influencing bicarbonate levels. Assistive devices for coughing or inhalations are also commonly used by pALS to improve ventilation and could also affect the acid–base balance, similarly to the use of NIV described in our cohort in Table S1. Furthermore, we may have included a selection bias by excluding patients with respiratory onset. However, this decision is justified by the rapid functional decline of patients with respiratory onset, and the potential bias their disease onset may introduce to our analysis. We also wanted to align with the previous study from Alarcan et al. (2023) to better compare our findings to their study. Finally, our study differed pALS by onset, which gives information about spreading patterns but does not specifically give information about the bulbar status.

Regarding clinical application, this study aimed to investigate the potential of serum bicarbonate as a marker for respiratory decline and survival among pALS, as to date it is not considered (Sun et al. 2020). VBG is an accessible and unexpensive tool used for routine assessments, which could provide vital screening information, does not require further organization, and can unlike VC measurements be performed independently of ALS phenotype and severity. This study should be regarded as an effort toward more equitable consideration and an incitement to further investigate alternatives to the existing eligibility criteria. Whether the use of serum bicarbonate as an alternative eligibility criterion to VC in ALS trials could be applied to all patients or specifically to patients with a bulbar onset remains needs to be confirmed in future studies. However, the correlation between VC and venous bicarbonate is the same for all patient groups (Table 2), suggesting that using venous bicarbonate as a surrogate would not negatively impact bulbar nor spinal pALS. This highlights the potential of venous bicarbonate to be less discriminative against bulbar pALS.

Further investigations need to confirm the equal performance of spirometry and venous bicarbonate and supplementary VBG parameters. A longitudinal study would be preferred, allowing the assessments throughout the progression of the disease. Increasing the sample size is also crucial to increase the statistical certainty and to obtain more conclusive results both for the general pALS population, but also for smaller subgroups such as pALS with respiratory or cognitive onset.

## 5 Conclusion

Venous bicarbonate appears to be a prognostic biomarker for survival and allows us to differentiate between pALS of spinal and bulbar onset. This cheap and easily accessible measure does not require training and could potentially be an alternative for VC used as a trial eligibility criterion.

## Author Contributions

**Juliette Foucher**: conceptualization, writing – original draft, methodology, writing – review and editing, formal analysis, project administration, data curation, software. **Therese Wellander**: conceptualization, writing – original draft, methodology, writing – review and editing, software, formal analysis, project administration, data curation. **Anikó Lovik**: supervision, writing – review and editing, methodology, software. **Linn Öijerstedt**: methodology, writing – review and editing, supervision. **Alexander Juto**: methodology, writing – review and editing, supervision. **Fang Fang**: methodology, writing – review and editing, supervision, funding acquisition. **Caroline Ingre**: investigation, funding acquisition, methodology, writing – review and editing, project administration, supervision.

## Conflicts of Interest

A.J. is a scientific advisory board member for Abilion Medical Systems; outside submitted work. C.I. has consulted for Cytokinetics, Pfizer, BioArctic, Novartis, Tikomed, Ferrer, Amylyx, Prilenia, Mitsubishi, and Abbvie. She is also a board member of Tobii Dynavox; all outside the submitted work. All other authors declare no conflicts of interest.

### Peer Review

The peer review history for this article is available at https://publons.com/publon/10.1002/brb3.70570.

## Supporting information



Supplementary Table 1. Participants survival and NIV characteristics, with repeated measures.
Supplementary Table 2. Characteristics of the study participants
Supplementary Figure 1. Kaplan Meier analysis on NIV use, with pALS stratified by venous bicarbonate quartiles. Follow up: diagnosis to NIV.
Supplementary Table 3. Factors influencing NIV use following an ALS diagnosis

## Data Availability

The dataset is available from the corresponding author upon reasonable request.
