# Supplementary material for: Venous Bicarbonate as a Prognostic Biomarker and Proposed Proxy for Vital Capacity to Be Used as an Eligibility Criterion in Amyotrophic Lateral Sclerosis Clinical Trials
Source: Brain Behav. 2025 May 18;15(5):e70570. doi: 10.1002/brb3.70570 (PMC12086301; doi:10.1002/brb3.70570)
Supplement: Supplementary file 1 — Supplementary Table 1. Participants survival and NIV characteristics, with repeated measures. Supplementary Table 2. Characteristics of the study participants Supplementary Figure 1. Kaplan Meier analysis on NIV use, with pALS stratified by venous bicarbonate quartiles. Follow up: diagnosis to NIV. Supplementary Table 3. Factors influencing NIV use following an ALS diagnosis [file BRB3-15-e70570-s001.docx]

**Venous bicarbonate as a prognostic biomarker and proposed proxy for vital capacity to be used as an eligibility criterion in amyotrophic lateral sclerosis clinical trials**

Authors:

**Juliette Foucher^1,2,*^, Therese Wellander^1*^**, Anikó Lovik^3,4^, Linn Öijerstedt^1,2^, Alexander Juto^1,2^, Fang Fang^3^, Caroline Ingre^1,2^

^1^ Department of Clinical Neuroscience, Karolinska Institutet, Stockholm, Sweden

^2^ Department of Neurology, ME Neurology, Karolinska University Hospital, Stockholm, Sweden

^3^ Unit of Integrative Epidemiology, Institute of Environmental Medicine, Karolinska Institutet, Stockholm, Sweden

^4^ Methodology and Statistics Unit, Institute of Psychology, Leiden University, Leiden, The Netherlands

*** shared first authors**

**Corresponding author’s information:**

- Name: Juliette Foucher
- Phone: +46-73-323-9382
- Email: [juliette.foucher@ki.se](mailto:juliette.foucher@ki.se)
- Country: Sweden
- ORCID: 0000-0001-9026-0268

**Supplementary material**

| **Supplementary Table 1. Participants survival and NIV characteristics, with repeated measures** | | | |
| --- | --- | --- | --- |
| **Characteristics** | **Total pALS**  (n=117) | **Spinal Onset patients**  (n=79) | **Bulbar Onset patients**  (n=38) |
|  | **Deceased pALS within January 2019 – October 2023** | | |
|  | (n=81) | (n=52) | (n=29) |
| Disease duration, *median months (IQR)* | 34 (23-42) | 35 (26–44) | 27 (21–35) |
|  | **pALS starting NIV during January 2019 - July2022** | | |
|  | (n=16) | (n=7) | (n=9) |
| Diagnosis-NIV start interval, *median months (IQR)* | 6 (2-9) | 8 (4-11) | 3 (1-9) |
|  | **pALS with several FVC values with bicarbonate and ALSFRS-R value within 60 days** | | |
|  | (n=43) | (n=33) | (n=10) |
| Time interval between the two VC consultations, *median days (IQR)* | 159 (100-245) | 183 (105-239) | 138 (81-403) |
| *Time intervals as median (Interquartile Range).*  Abbreviations: pALS, people with ALS. NIV, Non-Invasive Ventilation. VC, Vital Capacity. ALSFRS-R, Amyotrophic Lateral Sclerosis Functional Rating Scale-Revised | | | |

| **Supplementary Table 2. Characteristics of the study participants** | | | |
| --- | --- | --- | --- |
| **Characteristics** | **Female**  (n=63) | **Male**  (n=54) | **p-value of difference between sex** |
| VC value at baseline in %, *mean (SD)* | 76.5 (3.1) | 74.1 (2.7) | 0.58 |
| Venous bicarbonate at baseline in *mmol/L*, *mean (SD)* | 25 (0.3) | 24.7 (0.3) | 0.47 |
| ALSFRS-R score at baseline, *mean (SD)* | 33.7 (1.1) | 34.2 (1.1) | 0.76 |
| Abbreviations: ALSFRS-R, Amyotrophic Lateral Sclerosis Functional Rating Scale-Revised. VC, Vital Capacity. | | | |

| 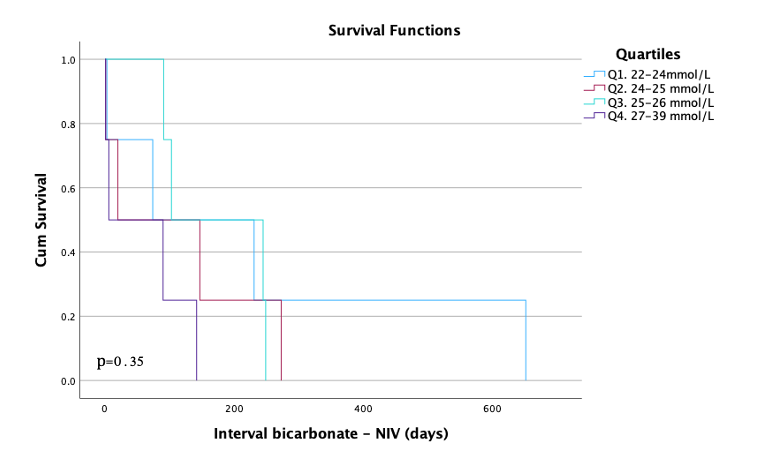 |
| --- |
| **Supplementary Figure 1. Kaplan Meier analysis on NIV use, with pALS stratified by venous bicarbonate quartiles**. Follow up: diagnosis to NIV. |

| **Supplementary Table 3. Factors influencing NIV use following an ALS diagnosis** | | | | |
| --- | --- | --- | --- | --- |
|  | **Univariable analysis** | | **Multivariable analysis** | |
|  | HR [95% CI] | *p*-value | HR [95% CI] | *p*-value |
| Venous bicarbonate | 1.19 [1.00-1.41] | 0.047 | 1.14 [0.96-1.35] | 0.148 |
| VC | 0.97 [0.94-0.99] | 0.042 | 0.97 [0.94-1.00] | 0.086 |
| Sex | 0.46 [0.16-1.33] | 0.150 | 0.42 [0.14-1.30] | 0.132 |
| Onset form | 1.52 [0.54-4.29] | 0.423 |  |  |
| ΔALSFRS-R | 1.15 [0.80-1.65] | 0.452 |  | |
| Age at diagnosis | 1.01 [0.96-1.07] | 0.626 |  | |
| *Uni- and multivariable Cox proportional hazards model for pALS at baseline. Results are presented as Hazard Ratios (95% Confidence Interval) and p-value. Statistical significance set at 0.2 for the univariable analysis.*  Abbreviations: ALSFRS-R, Amyotrophic Lateral Sclerosis (ALS) Functional Rating Scale-Revised. VC, Vital Capacity. ΔALSFRS-R, rate of functional decline over time. HR, hazard ratios. | | | | |
